# Interactions Between *Lr67* or *Lr34* and Other Leaf Rust Resistance Genes in Wheat (*Triticum aestivum*)

**DOI:** 10.3389/fpls.2022.871970

**Published:** 2022-05-20

**Authors:** Brent D. McCallum, Colin W. Hiebert

**Affiliations:** Agriculture and Agri-Food Canada, Morden Research and Development Centre, Morden, MB, Canada

**Keywords:** resistance, pyramid, gene, combinations, interaction, durable

## Abstract

The wheat multi-pest resistance genes *Lr67* and *Lr34* are similar in that they both condition resistance to many diseases, in a non-race-specific manner, and code for cellular transporters. *Lr34* plays a critical role in breeding wheat for disease resistance in large part because it interacts with other resistance genes to result in effective and durable resistance. To determine if *Lr67* interacts with other resistance genes in a similar manner as *Lr34* six different doubled haploid populations were developed which segregated for either *Lr67* or *Lr34* along with a second resistance gene, either *Lr13*, *Lr16*, or *Lr32*. The presence or absence of each of these genes in the progeny lines was determined by molecular marker analysis. These six populations were tested for leaf rust field resistance in the same environments to compare the effects of *Lr34* and *Lr67* alone, and in combination with *Lr13*, *Lr16* or *Lr32*. *Lr67* and *Lr34* significantly reduced the levels of rust severity, *Lr34* showed a significant interaction with *Lr13* but *Lr67* did not. Both genes interacted with *Lr16*, and *Lr67* had a significant interaction with *Lr32*. This analysis demonstrates the similar effect of *Lr67*, as seen with *Lr34*, on the interaction with other resistance genes to give a better level of resistance than with single resistance genes. While *Lr67* is not widely deployed in agriculture, it could play an important role in disease resistance in future wheat cultivars.

## Introduction

Wheat leaf rust is a very common and destructive disease of wheat internationally (Huerta-Espino et al., 2011) and in Canada (McCallum et al., 2016). Genetic resistance has proven effective in controlling this disease, however the *Puccinia triticina* Eriks. pathogen population has evolved virulence for most of the race-specific resistance genes widely deployed in wheat cultivars (McCallum et al., 2016). The race non-specific resistance gene *Lr34* has been used widely over many years in wheat cultivars and has remained effective. Canadian wheat cultivars commonly carry *Lr34* (McCallum et al., 2011) and it is frequently present in other wheat cultivars throughout the world. It also confers resistance to other diseases including stripe rust (*Yr18*, Singh, 1992), stem rust (*Sr57*, Dyck et al., 1985; Hiebert et al., 2010), powdery mildew (*Pm38*, Spielmeyer et al., 2005), and virus diseases (*Bdv1*, Singh, 1993).

One important feature of *Lr34* is that it interacts with other leaf rust resistance genes to give better levels of resistance. Ezzahiri and Roelfs (1989) determined that the durable and effective adult plant resistance in Era wheat was controlled by the interaction of *Lr13* and *Lr34*. These authors crossed plants of Era with the susceptible cultivar Baart, and they tested 473 and 367 F_3_ derived lines in the F_4_ generation in Minnesota USA and Morocco, respectively. They found that *Lr34* significantly enhanced the level of resistance conditioned by *Lr13* but the effect of *Lr34* on its own was not detected.

German and Kolmer (1992) crossed the Thatcher-*Lr34* near-isogenic line (NIL) with other Thatcher NILs containing different resistance genes. From F_2_ families ten to 16 plants with the lowest infection type when inoculated with *P. triticina* race 1 were selected and grown to maturity. Seed from these selected F_2_ plants was then grown in a rust nursery and the five or six most resistant F_3_ lines, homozygous for *Lr34* were selected and harvested. Two homozygous F_4_ lines per cross were tested as seedlings in the greenhouse and three to four plants of one F_4_ line were tested as adults in greenhouse tests. Selected lines with *Lr34* and a second leaf rust resistance gene were also field tested over two years. These authors found that *Lr34* enhanced resistance, both at the seedling and adult plant stages, in combination with many effective resistance genes, and this also resulted in lower adult plant infection types and leaf rust severity levels in the field. However, combinations involving *Lr34* with less effective or ineffective genes had the same level of resistance as Thatcher-*Lr34*.

Kloppers and Pretorius (1997) investigated two gene combinations of *Lr13*, *Lr34*, and *Lr37*. For glasshouse studies, they used a single F_4_ line from crosses between the pairs of Thatcher isolines that contained each of these resistance genes. These same F_4_ lines and six to eight sister lines from the same crosses were compared in field trials. They found that the two gene lines generally had better resistance as measured by latent period, field resistance, and the microscopic development of fungal structures. Interestingly, they also noted significant variation between sister lines for the *Lr34* + *Lr13* gene combination in which partial resistance was found in field trials. No variation was found among the sister lines from the gene combinations involving *Lr37* since the level of resistance was nearly complete.

The wheat leaf rust resistance gene *Lr67* is also race non-specific, is only effective at the adult plant stage, and confers multi-pest resistance to stripe rust (Hiebert et al., 2010; Herrera-Foessel et al., 2011) along with stem rust and powdery mildew like *Lr34* (Herrera-Foessel et al., 2014). Both *Lr34* and *Lr67* have been cloned and code for different types of cellular transporters (Krattinger et al., 2009; Moore et al., 2015). Mutations in either gene resulted in mutants that were susceptible to leaf, stem, and stripe rust (Spielmeyer et al., 2013). With combinations of most leaf rust resistance genes, the severity of disease observed is similar to the most effective of the genes involved. However, *Lr34* interacts with other leaf rust resistance genes, and combinations of genes involving *Lr34* are more resistant than any of the genes involved.

Some of the most common leaf rust resistance genes in Canadian wheat are *Lr2a*, *Lr10*, *Lr13*, *Lr14a*, *Lr16*, *Lr21*, and *Lr34* (McCallum et al., 2016). In this study, we choose to determine the interactions between both *Lr67* and *Lr34* with each of the genes; *Lr13*, *Lr16*, and *Lr32*. They represent a range of effectiveness from mostly ineffective (*Lr13*) to highly effective (*Lr32*). Both *Lr13* and *Lr16* are in many Canadian wheat cultivars, such as Carberry (Bokore et al., 2022), because popular wheat cultivars grown in the recent past like AC Barrie and AC Domain have either or both genes and donated these genes to the current generation of wheat cultivars. *Lr13* is relatively ineffective against the Canadian population of *P. triticina*, as nearly all isolates are virulent to *Lr13* (McCallum et al., 2021). However, it may still have an effect on reducing leaf rust severity in combination with other genes, such as *Lr34* and *Lr67*. Complete virulence to *Lr16* is rare in Canada (McCallum et al., 2021) but nearly all isolates have an intermediate level of virulence and combinations of *Lr16* with genes, such as *Lr34* and *Lr46*, are fairly effective at reducing leaf rust severity (Bokore et al., 2022). In contrast, *Lr32* is a very effective leaf rust resistance gene in Canada with no virulence detected to date (McCallum et al., 2021); however, it has not yet been deployed in any Canadian wheat cultivars. The Thatcher near-isogenic lines containing *Lr13* (RL4031), *Lr16* (RL6005), *Lr32* (RL6086), *Lr34* (RL6058), and *Lr13* + *Lr34* (RL6114) had annual rust severity averages in inoculated nurseries in Manitoba Canada over the years 2003–2021 of 76.6, 65.6, 30.8, 23.2, and 10.3%, respectively, compare with Thatcher at 81.9% (B. McCallum unpublished).

Given the many similarities between *Lr67* and *Lr34*, the objective of this study was to determine if *Lr67* also interacts with other resistance genes. To test this we developed six doubled haploid populations from the crosses with either single gene lines with *Lr34* or *Lr67* and each of the near-isogenic lines with either *Lr13*, *Lr16*, or *Lr32*. Each progeny line was genotyped with molecular markers to determine if the line had the resistant or susceptible allele of each gene involved in the population, except for *Lr13* which was determined by rust testing at the adult plant stage. Progeny from these crosses were field tested over four years to determine the resistance level of lines in each phenotypic class; susceptible, those having the resistant allele for either *Lr34* or *Lr67* alone, those only having the resistant allele of the second resistance gene (*Lr13*, *Lr16* or *Lr32*), and those with both genes.

## Materials and Methods

### Populations

Doubled haploid (DH) populations were developed from the crosses between Thatcher near-isogenic lines with *Lr34* (RL6058), *Lr67* (RL6077), *Lr13* (RL4031), *Lr16* (RL6005), and a Katepwa backcross line with *Lr32* (BW196R). The progeny populations consisted of 78 lines (Thatcher-*Lr13*/Thatcher-*Lr34*), 74 lines (Thatcher-*Lr13*/Thatcher-*Lr67*), 58 lines (Thatcher-*Lr16*/Thatcher-*Lr34*), 85 lines (Thatcher-*Lr16*/Thatcher-*Lr67*), 114 lines (Thatcher-*Lr34*/BW196R), and 113 lines (Thatcher-*Lr67*/BW196R). DH populations were generated using the maize pollination described by Thomas et al. (1997) except a single dicamba (100 ppm) treatment was used by placing a large drop with a syringe between the primary and secondary florets (all other florets were removed from each spikelet prior to emasculation) the day after pollination.

### Marker Analysis

To classify the progeny for the presence or absence of the genes targeted in each population, DNA markers were used to classify *Lr34*, *Lr32*, *Lr16*, and *Lr32*. The *Lr34* locus was genotyped using a PCR marker, caIND11, that targets an indel in the *Lr34* gene sequence (Dakouri et al., 2010). Both *Lr67* and *Lr16* were classified based on closely linked SNP markers, csSNP856 (Forrest et al., 2014) and kwm742 (Kassa et al., 2017) respectively. SSR markers wmc43 and barc135 were used to detect the presence of *Lr32* (Thomas et al., 2010). PCR products for caIND11 and SSR markers for *Lr32* were resolved using an ABI 3100 genetic analyzer (Applied Biosystems) as described by Somers et al. (2004). To genotype the SNP markers for *Lr67* and *Lr16*, KASP assays were performed as described by Kassa et al. (2016).

Given that current markers for *Lr13* are not tightly linked, *Lr13* was classified based on indoor leaf rust assays. For both populations that segregated for *Lr13*, two plants per line were grown to the adult plant stage in the greenhouse then inoculated with the *Lr13* avirulent *P. triticina* isolate 1–1 BBBD. While these populations also segregated for *Lr34* or *Lr67*, the presence of *Lr13* resulted in a clear and highly resistant reaction phenotype (‘;1-‘infection type as described by McCallum et al., 2021) that was not seen in those lines with *Lr34* or *Lr67* or susceptible lines which had more susceptible pustule types. Therefore all lines could therefore be scored as having either the resistant or the susceptible allele for *Lr13*.

### Leaf Rust Field Resistance

These populations were grown in leaf rust inoculated, irrigated, field nurseries at Morden Manitoba during four years 2012–2015, with two replications per year, except in 2012 in which a single row was planted for each line. Progeny from the populations lines Thatcher-*Lr13*/Thatcher-*Lr34* and Thatcher-*Lr13*/Thatcher-*Lr67* were tested for an additional two field seasons in 2016 and 2017, with two replicates per season. Each line was seeded in approximately 1 m rows. Spreader rows of susceptible wheat were planted at regular intervals to help the epidemic develop and infect the test lines. Spreader rows were inoculated a few times each year with a mixture of urediniospores in Soltrol mineral oil. The inoculum was a mixture of *P. triticina* virulence phenotypes, representative of those found in western Canada the previous year (McCallum et al., 2018, 2019, 2020, 2021). The lines were assessed for the level of leaf rust infection on the flag leaves using a 0–100% modified Cobb scale (Peterson et al., 1948). They were also assessed for pustule type (R-MR-MS-S) but only the severity data were used for analysis since this was a better measure of the proportion of the flag leaves infected with leaf rust.

Leaf rust severity percentage ratings were converted to proportions for analysis, then back to percentages for presentation. Data from each population were analyzed separately with SAS 9.3 (SAS Institute Inc.) using PROC GLIMMIX (beta distribution) with the presence or absence of the genes, and their interaction, in each progeny line of the population as dependent variables and replication within each year as the random variable. Within each population, the groups of lines with all the possible gene combinations were compared pairwise to each other using LSMEANS.

## Results

**Figure 1:**
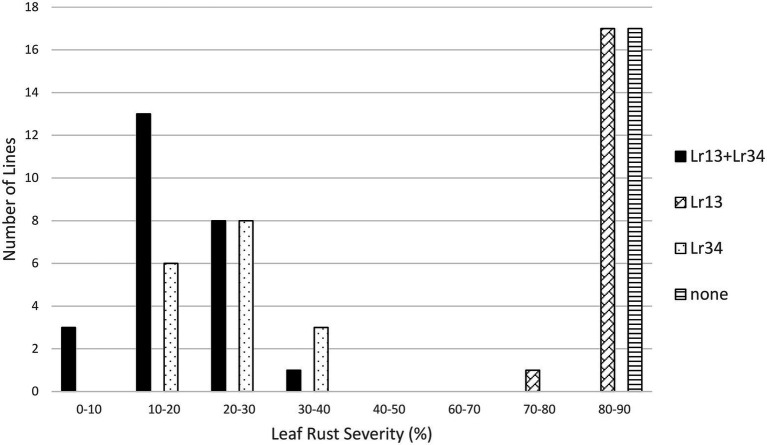
Average leaf rust field severity (2012–2017) for progeny lines from the cross *Lr13*/*Lr34*.

**Figure 2:**
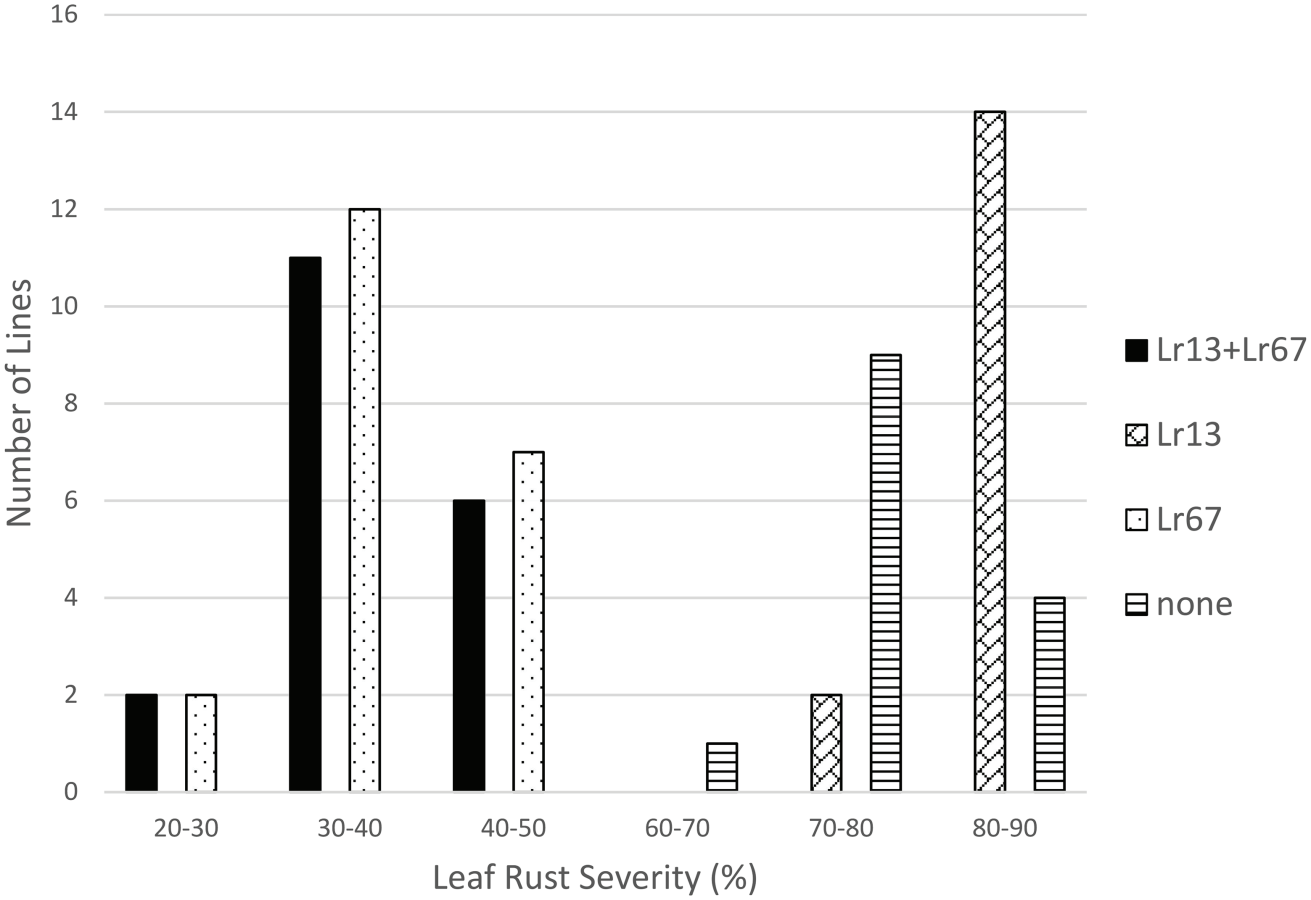
Average leaf rust field severity (2012–2017) for progeny lines from the cross *Lr13*/*Lr67*.

The effects of both *Lr13* and *Lr34* were significant in the *Lr13/Lr34* population, as was the interaction between *Lr13* and *Lr34* (Table 1). The lines that had both genes had the lowest level of leaf rust severity (17.7%), followed by lines with only *Lr34* (23.8%), lines with only *Lr13* (84.3%), and lines with neither gene (85.7%) (Table 1; Figure 1). When these four classes of lines were compared against each other, each class was significantly different from the others at the *p* < 0.01 level, except lines with only *Lr13* which were not different from lines with neither gene (Table 2). In the *Lr13/Lr67* population, the effect of *Lr67* was significant, but not that of *Lr13*. In contrast to the *Lr13/Lr34* population, there was no significant interaction between the two genes. In this population, lines with both genes had a similar level of leaf rust severity (36.2%) compared to lines with only *Lr67* (37.0%) and lines that only had *Lr13* were similar (80.5%) with lines that had neither gene (79.0%) (Tables 1, 2; Figure 2).

**Table 1:** Effect of each gene and their interaction on the severity of leaf rust and the average severity for each genotypic class.

Population	Gene A		Gene B		Interaction		Average Leaf Rust Severity and Standard Error (%)			
A/B^a^	*F* Value	Pr > F	F Value	Pr > F	F Value	Pr > F	RA/RB	RA/SB	SA/RB	SA/SB
*Lr13/Lr34*	18.74	<0.0001	2938.98	<0.0001	5.78	0.0164	17.7 (2.0)	84.3 (1.9)	23.8 (2.5)	85.7 (1.8)
*Lr13/Lr67*	0.41	0.5233	1785.96	<0.0001	2.0	0.1574	36.19 (4.0)	80.5 (2.7)	37.0 (4.0)	79.0 (2.9)
*Lr16/Lr34*	5.93	0.0154	946.57	<0.0001	16.1	<0.0001	24.5 (1.7)	82.2 (1.2)	34.7 (1.7)	80.3 (1.7)
*Lr16/Lr67*	22.98	<0.0001	1068.38	<0.0001	19.21	<0.0001	38.0 (1.5)	82.0 (1.1)	50.1 (1.6)	82.3 (1.0)
*Lr32/Lr34*	472.02	<0.0001	1132.18	<0.0001	1.28	0.2579	3.1 (0.7)	38.1 (4.5)	16.9 (2.7)	82.6 (2.7)
*Lr32/Lr67*	541.34	<0.0001	690.83	<0.0001	9.33	0.0023	2.1 (0.5)	29.7 (3.9)	23.6 (3.3)	77.0 (3.3)

a *Gene A is the first gene listed and gene B is the second gene. “R” indicates the resistant allele at this locus, and “S” indicates the susceptible allele*.

**Figure 3:**
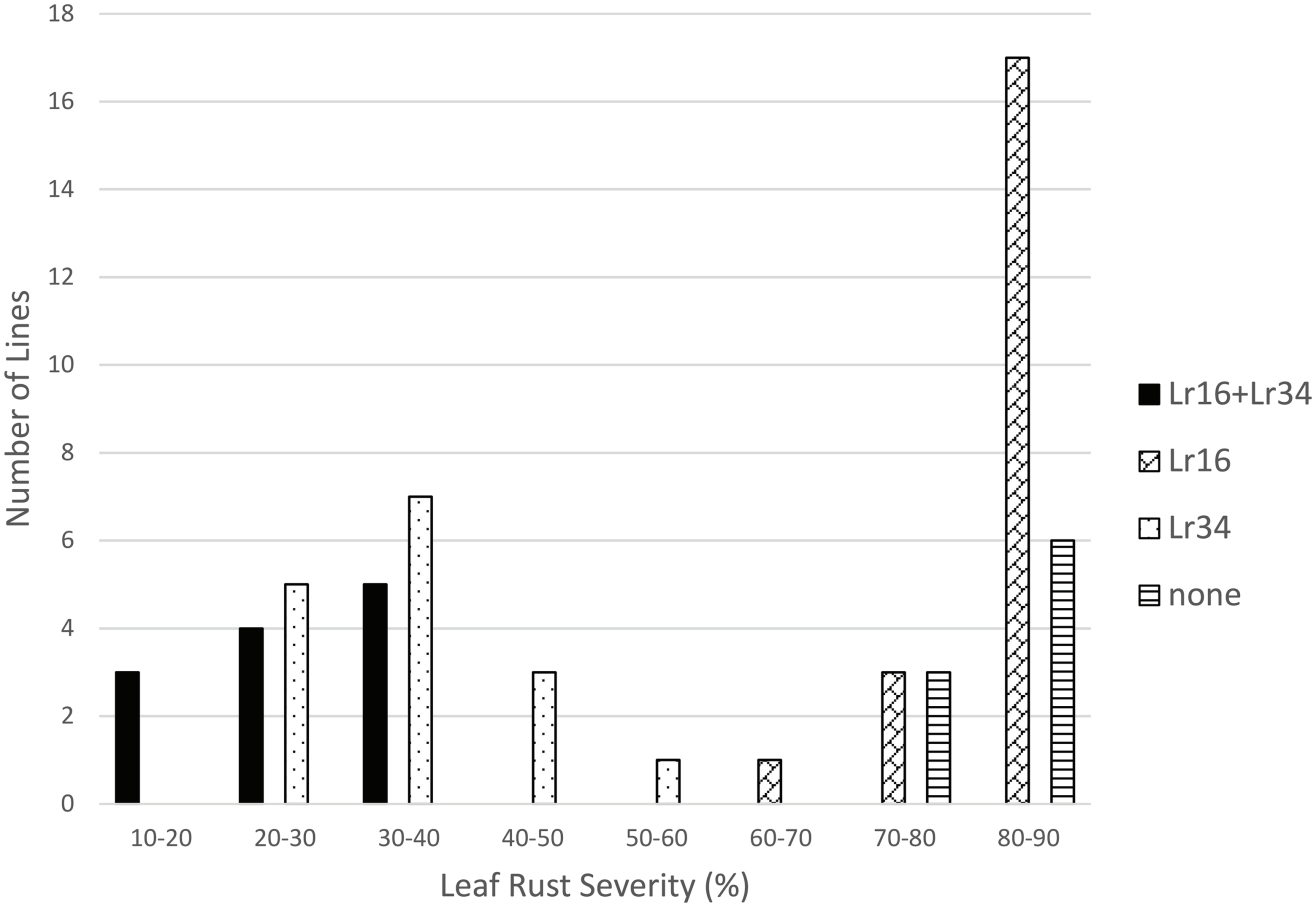
Average leaf rust field severity (2012–2015) for progeny lines from the cross *Lr16*/*Lr34*.

**Figure 4:**
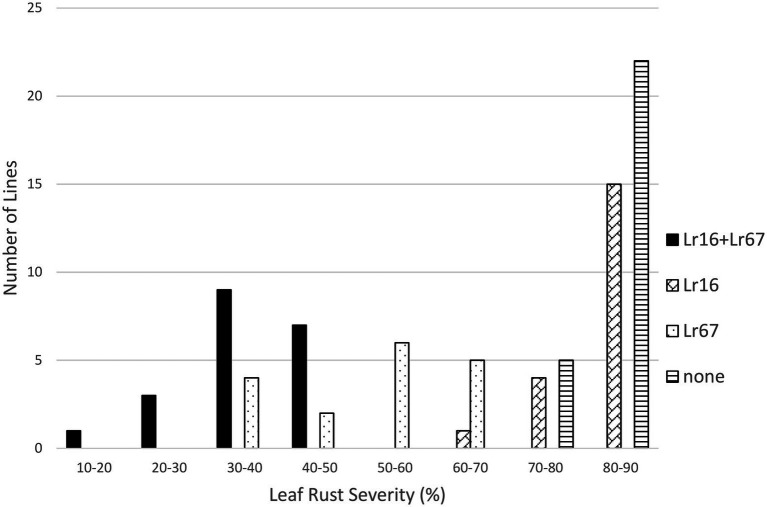
Average leaf rust field severity (2012–2015) for progeny lines from the cross *Lr16*/*Lr67*.

Both *Lr16* and *Lr34* were significant in reducing leaf rust severity in the *Lr16/Lr34* population, and the interaction between the genes was also significant (*p* < 0.01). Lines with both genes had the lowest leaf rust severity (24.6%), followed by lines with only *Lr34* (34.7%), lines with only *Lr16* however had a similar level of leaf rust severity (82.2%) as the lines with neither gene (80.3%) (Tables 1, 2; Figure 3). Similarly, in the *Lr16/Lr67* population, both *Lr16* and *Lr67* were significant in reducing leaf rust severity, and their interaction was significant (*p* < 0.01). Again lines with both genes had the lowest level of severity (38.0%), followed by lines with only *Lr67* (50.1%) and lines with only *Lr16* were similar (82.0%) to lines with neither gene (82.3%) (Tables 1, 2; Figure 4).

**Figure 5:**
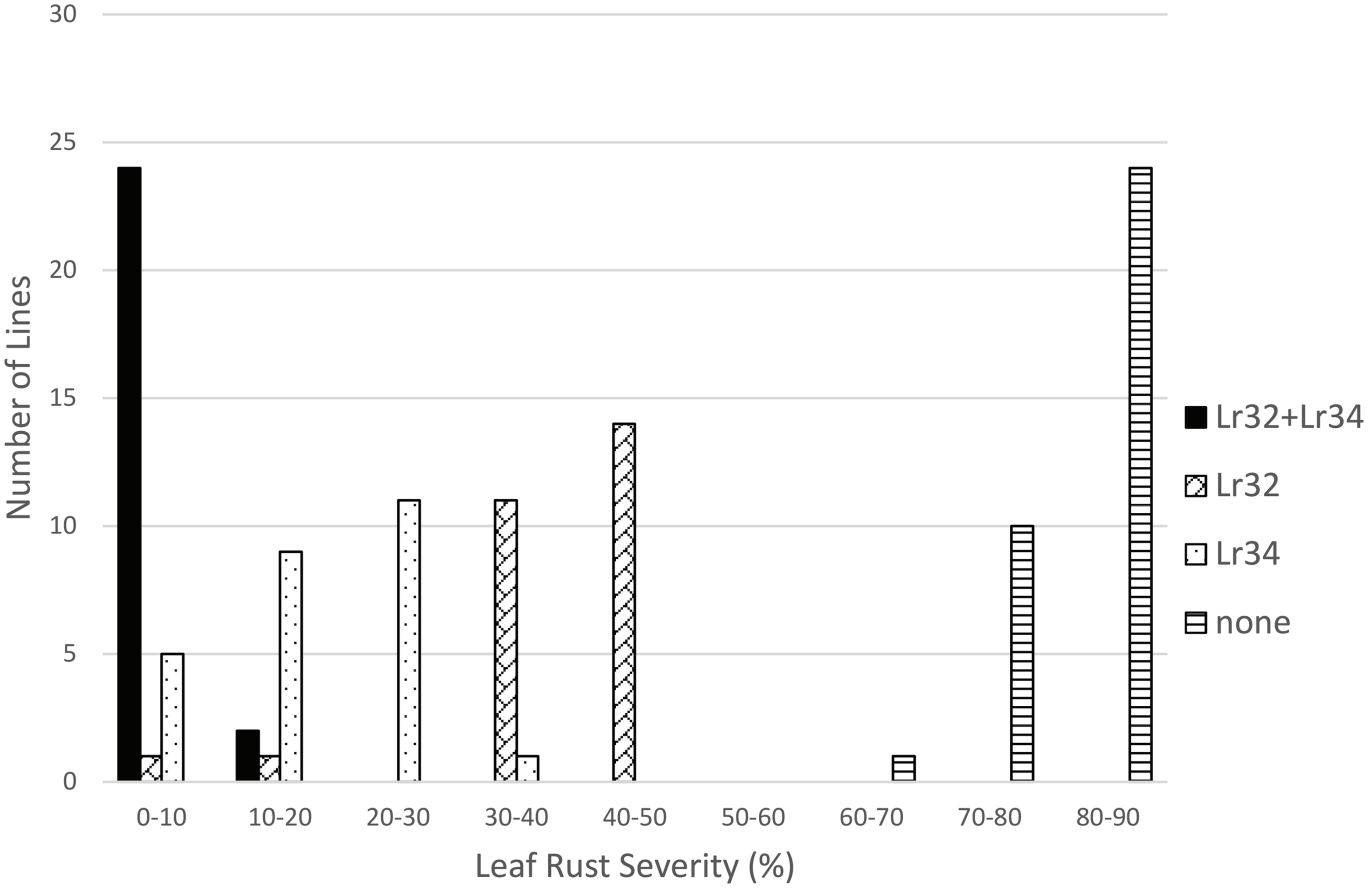
Average leaf rust field severity (2012–2015) for progeny lines from the cross *Lr32*/*Lr34*.

**Figure 6:**
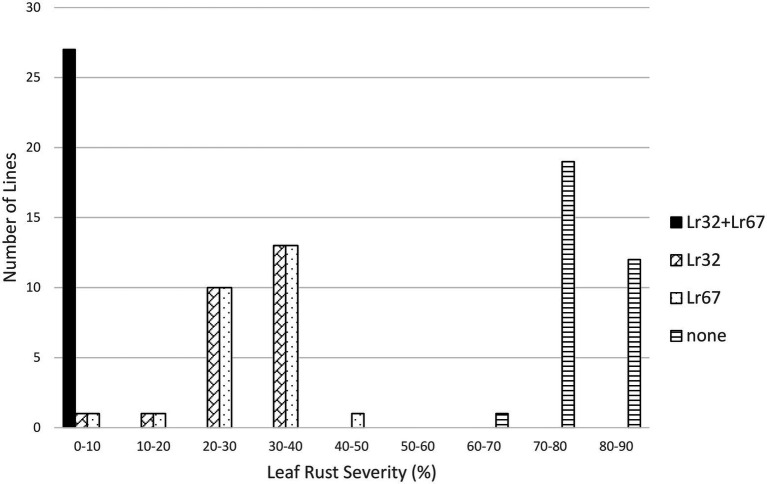
Average leaf rust field severity (2012–2015) for progeny lines from the cross *Lr32*/*Lr67*.

In the *Lr32/Lr34* population, both genes significantly reduced leaf rust severity (*p* < 0.01); however, their interaction was not significant. Lines with both genes were very resistant (3.1%), followed by lines with only *Lr34* (16.9%) or only *Lr32* (38.1%), compared to lines with neither gene (82.6%) (Table 1; Figure 5). However, each class of lines was significantly different from the other classes (Table 2). Similarly for the *Lr32/Lr67* population both genes significantly reduced leaf rust severity (*p* < 0.01), their interaction was however significant (*p* < 0.01). Again lines with both genes had the lowest leaf rust severity (2.1%), followed by those with only *Lr67* (23.6%), those with only *Lr32* (29.7%), and those with neither gene (77.0%) (Table 1; Figure 6). Each class of line was also significantly different from all the other classes (Table 2).

**Table 2:** Significance of the difference between groups of lines with different combinations of resistance genes.

Population	RA/RB vs.	RA/RB vs.	RA/RB vs.	RA/SB vs.	RA/SB vs.	SA/RB vs.
**A/B** ^**a**^	**RA/SB**	**SA/RB**	**SA/SB**	**SA/RB**	**SA/SB**	**SA/SB**
*Lr13/Lr34*	<0.0001	<0.0001	<0.0001	<0.0001	0.2101	<0.0001
*Lr13/Lr67*	<0.0001	0.5239	<0.0001	<0.0001	0.1959	<0.0001
*Lr16/Lr34*	<0.0001	<0.0001	<0.0001	<0.0001	0.3063	<0.0001
*Lr16/Lr67*	<0.0001	<0.0001	<0.0001	<0.0001	0.7872	<0.0001
*Lr32/Lr34*	<0.0001	<0.0001	<0.0001	<0.0001	<0.0001	<0.0001
*Lr32/Lr67*	<0.0001	<0.0001	<0.0001	0.0002	<0.0001	<0.0001

a *Gene A is the first gene listed and gene B is the second gene. “R” indicates the resistant allele at this locus, and “S” indicates the susceptible allele*.

## Discussion

This study compared how *Lr34* and *Lr67* interact in combination with other resistance genes in progeny populations. They were each paired with *Lr13*, *Lr16*, or *Lr32* in populations segregating for one of those genes and either *Lr34* or *Lr67*. In the populations that segregated for *Lr13*, the effect of *Lr13* was significant in the population with *Lr34* but not with the population involving *Lr67*. There was a significant interaction between *Lr13* and *Lr34* whereas there was no significant interaction between *Lr13* and *Lr67*. It appears that *Lr34* and *Lr67* differ in their interaction with *Lr13*. The lack of interaction with *Lr67* may reflect the marginal resistance provided by *Lr13* and that fact that most of the virulence phenotypes in Canada are virulent on *Lr13*. During the years of the field tests the frequency of virulence to *Lr13* in the population was close to 100% (McCallum et al., 2018, 2019, 2020, 2021). Both *Lr34* and *Lr67* were effective in reducing the leaf rust severity, but *Lr34* had a larger effect on reducing leaf rust compared to *Lr67* which may reflect a different in their interactive magnitudes. The *Lr13* + *Lr34* gene combination appeared to be more effective than either gene alone in this study, and in previous studies (Ezzahiri and Roelfs, 1989; German and Kolmer, 1992; Kloppers and Pretorius, 1997).

The resistance gene *Lr16* was more effective than *Lr13* in reducing the severity of leaf rust. While the frequency of virulence to *Lr16* is very low (near 0%) (McCallum et al., 2021), most isolates have an intermediate response to *Lr16* and the Thatcher-*Lr16* wheat line is fairly susceptible in field trials as the long term leaf rust severity average of the Thatcher isoline with *Lr16* was 65.6% compared with Thatcher at 81.9% (B. McCallum unpublished). Overall *Lr16* had a significant effect on leaf rust in these populations and had significant interactions with both *Lr34* and *Lr67* (Table 1). The effect of *Lr16* is mainly in its interaction with either *Lr34* or *Lr67*, because the lines with both *Lr16* and either *Lr34* or *Lr67* were significantly more resistant than lines with just *Lr34* or *Lr67*, however, the lines with *Lr16* alone were not significantly different from lines with neither gene in this study (Table 2). Both *Lr34* and *Lr67* interacted with *Lr16* to produce an enhanced resistance, even though lines with *Lr16* alone were not significantly different from susceptible lines. This may reflect on the ability of *Lr16* to interact with other resistance genes, as it has been shown previously to do with genes like *Lr13* (Samborski and Dyck, 1982) and both *Lr34* and *Lr46* in Carberry (Bokore et al., 2022).

This effect of enhancement was also seen with the populations involving *Lr32*. Alone *Lr32* was very effective in reducing the severity of leaf rust, as were both *Lr34* and *Lr67*. The lines with two gene combinations of *Lr32* + *Lr34* and *Lr32* + *Lr67* were even more resistant than any of these genes alone. Virulence has not been detected in Canada to *Lr32* and the Thatcher-*Lr32* line is moderately resistant in field trials. Both *Lr34* and *Lr67* had the ability to enhance the resistance of *Lr32* when in combination with this resistance gene. However, the interaction was only significant between *Lr32* and *Lr67* (Table 1). The parental line containing *Lr32* in these crosses also contained *Lr13*, which segregated in both populations, although its presence or absence was not determined it would have been distributed evenly between phenotypic classes. The effects of *Lr34* and *Lr67* were stronger in these populations, than in the other populations analyzed, which could reflect the fact that *Lr13* was segregating in these crosses and potentially interacting with the other leaf rust resistance genes.

Overall *Lr67* behaved similarly to *Lr34* in this study. Both genes consistently reduced the level of leaf rust in each population, *Lr67* did not interact with *Lr13*, which was only effective on its own in the *Lr34* population, however, both did have a significant interaction with *Lr16*. The effect of *Lr16* was significant overall but it appeared to be effective only when in combination with either *Lr34* or *Lr67* to result in a significantly lower level of leaf rust than with either gene alone. Both genes also significantly reduced leaf rust when combined with the effective resistance gene *Lr32*.

Interactions between race-specific leaf rust resistance genes and *Lr34* have been analyzed in previous studies. The studies by Kloppers and Pretorius (1997) and German and Kolmer (1992) both showed that lines carrying *Lr34* plus a race-specific gene had lower disease severity than either gene singly. The data presented here show the same trend. The most direct comparison between these three studies is the interaction between *Lr34* and *Lr13* as this combination was present in all of the studies. German and Kolmer (1992) report what appears to be the strongest interaction between *Lr13* and *Lr34*. However, there are some key differences between how these studies were conducted. German and Kolmer (1992) selected a single F_4_ line that was homozygous for *Lr34* and *Lr13* and they reportedly selected the most resistant homozygous line for field testing. This was also done for the other gene combinations in their study. Kloppers and Pretorius (1997) analyzed six lines with *Lr34* and *Lr13* and they found varying responses between lines. At the time of their final field rating, the severities of the six lines ranged from 10 to 50%. The interaction would look different if they had only used the most resistant line.

Similarly, in our study, DH lines carrying *Lr34* and *Lr13* had mean severities ranging from 7 to 37%. There was at least some range of severity levels among the progeny lines for all the various gene combinations generated in this study. While Thatcher near-isogenic lines were used primarily as the parental lines in this study, intercrossing these lines resulted in significant variation between lines with the same major gene combinations, similar to the variation found by Kloppers and Pretorius (1997) with the *Lr13* + *Lr34* sister lines. Using multiple lines or populations gives a more accurate representation of the interactions between genes as all of the other factors will also be segregating and correlated errors are minimized. In the present study, we also compared DH lines carrying each gene singly for the same reason as when the NILs were developed, the best phenotypes were selected which may not best represent the resistance conferred by the Lr gene in question.

Both *Lr34* and *Lr67*, along with a third multi-pest non-race-specific adult plant resistance gene *Lr46*, are important components of resistance in CIMMYT and Mexican wheat cultivars (Singh et al., 2008; Huerta-Espino et al., 2020). Deployed alone these adult plant multi-pest resistance genes did not confer adequate resistance, but combinations of 4–5 genes usually results in near immunity levels of resistance (Singh et al., 2011). Interestingly, *Lr67* was deployed in many Mexican wheat cultivars developed in the 1950s, but not in later cultivars due to chance parental selection in which only *Lr34* was used. Since the donor lines for *Lr34* (RL6058) and *Lr67* (RL6077) showed similar resistance phenotypes, both lines were initially thought to have *Lr34* and were used interchangeably as a source for *Lr34* in the 1950s by the CIMMYT wheat breeding program (Huerta-Espino et al., 2020).

Gene pyramids involving *Lr34* are also common in Canadian wheat cultivars (McCallum et al., 2011; Toth et al., 2018). The highly resistant Canadian cultivar Pasqua contains five resistance genes including *Lr34* (Dyck, 1993). *Lr34* appears to be key to its high level of resistance, as progeny lines derived from Pasqua with the four other resistance gene were fairly susceptible (McCallum and Thomas, 2014). Similarly, the high level of durable resistance in the cultivar Carberry is conditioned by the combination of *Lr2a*, *Lr16*, *Lr23*, *Lr13*, *Lr34*, and *Lr46* (Bokore et al., 2022), in which the key is the interaction of *Lr34* and *Lr46* with the other resistance genes. These multi-pest resistance genes along with others, such as *Sr2*, appear to function very well in combinations with other genes to condition effective and durable resistance, often by boosting the effect of other resistance genes (Ellis et al., 2014). Randhawa et al. (2018) found that *Lr34/Yr18/Sr57* interacted with *Lr68* to reduce leaf rust and *Sr2/Yr30* to reduce rust severity to leaf, stem, and stripe rust in a segregating population.

It appears that *Lr67* could play a similar and important role in leaf rust resistance, like *Lr34* or *Lr46*, if it was combined with other resistance genes, such as *Lr16* and *Lr32*, in which it could interact to result in lower levels of leaf rust severity and improved durability of resistance. However, *Lr67* failed to show the same significant interaction with *Lr13* that *Lr34* demonstrated, and the effect of *Lr34* alone was stronger than that of *Lr67* in each of the pairs of populations. In contrast, the interaction between *Lr32* and *Lr67* was significant whereas that between *Lr32* and *Lr34* was not. While *Lr67* was deployed in many CIMMYT wheat cultivars from the 1950s (Huerta-Espino et al., 2020), it is not deployed in Canadian wheat cultivars to date. If it was deployed in Canada and other countries it could improve the rust resistance and durability of future wheat cultivars.

## Data Availability Statement

The original contributions presented in the study are included in the article, further inquiries can be directed to the corresponding author.

## Author Contributions

BM and CH planned the experiments, analyzed the data, and co-wrote the manuscript. CH developed the populations analyzed in this study and conducted the molecular marker analysis of all progeny lines. BM conducted the leaf rust phenotyping of the populations. All authors contributed to the article and approved the submitted version.

## Funding

Funding for this project was from Agriculture and Agri-Food Canada.

## Conflict of Interest

The authors declare that the research was conducted in the absence of any commercial or financial relationships that could be construed as a potential conflict of interest.

## Publisher’s Note

All claims expressed in this article are solely those of the authors and do not necessarily represent those of their affiliated organizations, or those of the publisher, the editors and the reviewers. Any product that may be evaluated in this article, or claim that may be made by its manufacturer, is not guaranteed or endorsed by the publisher.
